# Malaria infection, disease and mortality among children and adults on the coast of Kenya

**DOI:** 10.1186/s12936-020-03286-6

**Published:** 2020-06-17

**Authors:** Alice Kamau, Grace Mtanje, Christine Mataza, Gabriel Mwambingu, Neema Mturi, Shebe Mohammed, Gerald Ong’ayo, Gideon Nyutu, Amek Nyaguara, Philip Bejon, Robert W. Snow

**Affiliations:** 1 KEMRI-Wellcome Trust Research Programme, Kilifi, Kenya; 2 Ministry of Health, Kilifi County Government, Kilifi, Kenya; 3 Centre for Tropical Medicine and Global Health, Nuffield Department of Clinical Medicine, University of Oxford, Oxford, UK

**Keywords:** Malaria, Age-pattern, Adults, Immunity, Infection, Mortality, Severe disease

## Abstract

**Background:**

Malaria transmission has recently fallen in many parts of Africa, but systematic descriptions of infection and disease across all age groups are rare. Here, an epidemiological investigation of parasite prevalence, the incidence of fevers associated with infection, severe hospitalized disease and mortality among children older than 6 months and adults on the Kenyan coast is presented.

**Methods:**

A prospective fever surveillance was undertaken at 6 out-patients (OPD) health-facilities between March 2018 and February 2019. Four community-based, cross sectional surveys of fever history and infection prevalence were completed among randomly selected homestead members from the same communities. Paediatric and adult malaria at Kilifi county hospital was obtained for the 12 months period. Rapid Diagnostic Tests (CareStart™ RDT) to detect HRP2-specific to *Plasmodium falciparum* was used in the community and the OPD, and microscopy in the hospital. Crude and age-specific incidence rates were computed using Poisson regression.

**Results:**

Parasite prevalence gradually increased from childhood, reaching 12% by 9 years of age then declining through adolescence into adulthood. The incidence rate of RDT positivity in the OPD followed a similar trend to that of infection prevalence in the community. The incidence of hospitalized malaria from the same community was concentrated among children aged 6 months to 4 years (i.e. 64% and 70% of all hospitalized and severe malaria during the 12 months of surveillance, respectively). Only 3.7% (12/316) of deaths were directly attributable to malaria. Malaria mortality was highest among children aged 6 months–4 years at 0.57 per 1000 person-years (95% CI 0.2, 1.2). Severe malaria and death from malaria was negligible above 15 years of age.

**Conclusion:**

Under conditions of low transmission intensity, immunity to disease and the fatal consequences of infection appear to continue to be acquired in childhood and faster than anti-parasitic immunity. There was no evidence of an emerging significant burden of severe malaria or malaria mortality among adults. This is contrary to current modelled approaches to disease burden estimation in Africa and has important implications for the targeting of infection prevention strategies based on chemoprevention or vector control.

## Background

Parasite exposure, age and immunity are intimately related [1]. In malaria, it is believed that frequent parasite exposure from birth first leads to the acquisition of functional immunity against severe disease and death, followed by immunity to mild, self-limiting disease, and finally to the ability to regulate infection per se (i.e. anti-parasitic immunity) [2–5]. The age at which these features of immunity occur depends on the quantity and timing of repeat infections and consequently varies in space and time [6–9].

*Plasmodium falciparum* continues to infect millions of people in sub-Saharan Africa (SSA) every year [10, 11]. The balance between the direct and indirect fatal consequences of malaria infection remain poorly defined [12]. Direct attribution of malaria as a cause of death is fraught with problems, as most deaths occur outside of health facilities with the ability to provide a parasitological diagnosis and depends mostly on histories of symptoms provided by bereaved relatives [13]. Surveys of malaria infection prevalence are common epidemiological investigations of disease but mortality incidence are much less common and both often focus on young children [6, 14–17].

The components of malaria surveillance, as articulated in one of the first textbooks on malaria, emphasized the importance of defining infection and morbidity among all age groups, enabling a complete understanding of risks within a community [18]. Contemporary examples of coincidental descriptions of the epidemiology of infection and disease in all age groups are rare [19–30].

The paucity of empirical data on the relationship between malaria infection, disease and mortality outcomes across all age groups hinders both the understanding of age-dependent stages of immunity and the reliable and complete description of malaria’s contribution to the burden of disease in Africa. Here, an epidemiological investigation of parasite prevalence, the incidence of fevers associated with infection, severe hospitalized disease and mortality among a population aged older than 6 months on the Kenyan coast is presented to define patterns of malaria infection to death.

## Methods

### Study area

In 2000, a longitudinal Kilifi health and demographic surveillance system (KHDSS) was initiated in an area north and south of Kilifi creek along the coast of Kenya, surrounding the Kilifi County Hospital [31]. The area is subdivided into 186 enumeration zones (EZ) each with approximately 226 homesteads per EZ. Each household, major landmark and footpath has been mapped using Geographic Positioning Systems. Fieldworkers visit every household three times a year to monitor births, deaths and migration events.

**Fig. 1:**
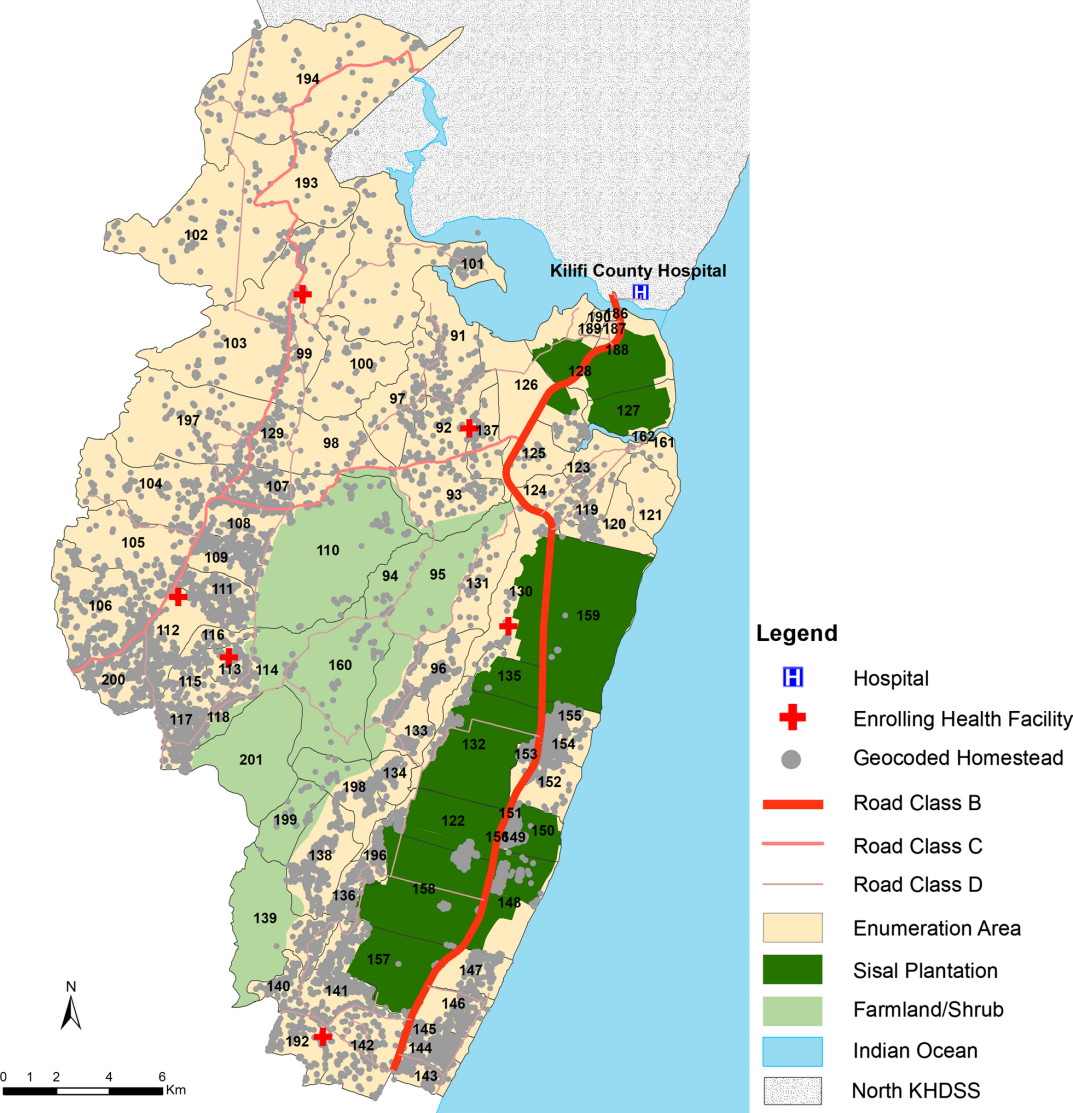
Map showing the study sites. This includes location of 29 geo-coded health facilities south of KHDSS including Kilifi county hospital (KCH) overlaid on the geocoded homesteads, location of the sisal plantation, farmlands or shrubs and the roads. Health facility where patients were enrolled are shown by the red crosses, blue cross are other health facilities in the area; enumeration zones are the cream polygons; road class means: B, National Trunk Road; C, Primary Roads; D, Secondary Roads

Kilifi has undergone marked changes in malaria transmission, areas in the North of the KHDSS have shown dramatic reductions to below 1% parasite prevalence [32]. Reductions have also occurred in the southern part of the KHDSS but this area continues to support low-moderate transmission. Transmission follows a bimodal seasonal pattern associated with rainy seasons typically occurring between April and June and between October and December. Areas in the southern KHDSS were selected within 2 km of six health facilities; the facilities were selected on the basis that they were rural public health facilities, with a high burden of patients (a minimum of 10 patients per day), and were not part of ongoing active surveillance [33] (Fig. 1).The area included 36 EZs covering 9596 homesteads and an enumerated mid-year population of 72,560 in 2018.

### Community-based parasite prevalence surveillance

The survey was conducted among members of randomly selected homesteads within the catchment area of the six health facilities. Sampling was conducted four times in 1 year i.e. in May–June 2018, August 2018, October 2018 and December 2018–January 2019, including 6479 participants in total.

A malaria rapid diagnostic test (RDT) (CareStart™) to detect histidine-rich protein (HRP2) specific to *P. falciparum* was performed on all consenting participants, irrespective of fever status. Participants with fever and/or a positive rapid test were advised to seek treatment at the nearest health facility.

### Health facility-based passive surveillance of fever infections

At each facility, the study included records of all patients ≥ 6 months of age that sought treatment between March 2018 and February 2019 with a history of fever in the last 24 h as part of presenting illness or a measured axillary temperature of ≥ 37.5 °C. All febrile patients of all ages were tested by a trained field worker using an RDT (CareStart™). If the RDT results were positive, the patient received appropriate treatment as per the Government of Kenya guidelines for malaria-case management [34].

### Clinical surveillance at Kilifi county hospital

Kilifi county hospital (KCH) is a level IV hospital and serves as the first-level referral centre for both adults and children from the study area, located between 5 and 32 km for the study residents (Fig. 1). Paediatric in-patient surveillance was as previously described [35]. Every child admitted to the hospital is investigated with a thick or thin blood smear for microscopy to ascertain *Plasmodium* infection, regardless of presentation. Adult in-patient surveillance was initiated in 2007 [36]. Thick or thin blood smear for malaria microscopy is only performed on febrile admissions. At discharge, medical staffs assigned a primary and/or secondary diagnosis based on all available data.

For both paediatric and adult admissions a diagnosis was deemed undetermined if there was insufficient laboratory or clinical evidence to assign a unique diagnosis. Malaria was defined as a primary clinical diagnosis with a confirmed positive malaria slide, taken at any time during the admission, and not including another major secondary clinical diagnosis, for example underlying sickle cell disease (SCD). Slide negative malaria or febrile convulsion was re-classified as an unspecified disease (UN) in the absence of a secondary diagnosis. Severe malaria was defined as per the WHO definition [37], and categorized as a positive malaria slide with: cerebral malaria (Blantyre Coma Score of < 3 among children and a Glasgow Coma Score of < 11 among adults); severe malaria anaemia (haemoglobin concentration < 5 gm/dl among children and < 7 gm/dl among adults); respiratory distress (deep acidotic breathing); multiple convulsions were recorded only among paediatric malaria admissions and defined as two or more convulsions in the 24-h period prior to admission. Other features of severe malaria, including hyperparasitaemia, haemoglobinuria (blackwater), jaundice, shock, pulmonary oedema, renal impairment and prostration, were not systematically documented or standardized between admission wards and were not considered further.

### Mortality surveillance and cause of death

Deaths among residents of the catchment areas were detected immediately if they occurred at KCH or during 4 monthly household re-enumerations. Causes of death (COD) among individuals aged ≥ 6 months were attributed through a multi-stage process. Causes were first attributed to the primary diagnosis assigned through clinical and laboratory investigations if the death occurred in hospital. If death occurred within 30 days post discharge from hospital, details of the discharge diagnoses were used to provide information related to the subsequent cause of death. If the deceased had had contact with the hospital during the preceding 24 months and was diagnosed with a chronic condition (e.g. HIV, SCD, cancer, diabetes, epilepsy) this was used to attribute the primary COD.

For all remaining deaths without hospital contact, a verbal autopsy (VA) tool was used as previously described [38–41]. Causes of deaths were inferred using the InterVA-4 model; an expert opinion-based algorithm that uses Bayes theorem [38, 42, 43]. The algorithm assigns at most three probable COD with corresponding likelihood. The secondary and tertiary causes are only reported if the likelihood is more than half of the most likely cause. If none of the causes has a probability > 0.4, then death is considered ‘indeterminate’. In this study area, the prevalence of malaria and HIV were considered to be high in the model in order to determine the likelihood of death being related to malaria or HIV. In addition, details provided in the free-text fields related to the primary and underlying COD were re-evaluated and SCD, assault, diabetes, HIV, road traffic accident (RTA)/trauma, known epilepsy, and suicide were regarded as unambiguous if the likelihood was 100% or from processing open text responses.

For individuals who had no contact with the hospital and for whom VA was not done, multiple imputation was used to assign a plausible cause of death based on the distribution of known causes [44]. The multivariate imputation by chained equations (MICE) algorithm was used to impute values as previously described [45]. MICE was conducted in R version 3.6.1 [R Core Team (2019), Vienna, Austria].

### Analysis

Chi-square test was used to compare difference in proportions and a logistic regression model was used to analyze the risk of malaria with adjustment for repeated measures. Adjusted odds ratios (aOR) quoted have been adjusted for factors specified in the text. In order to compute the period prevalence (defined using all cases testing RDT positive including re-attendance) and incidence rate (defined using records of first cases only of RDT positive patients), the study area’s person-years of observation from the KHDSS stratified by age was used. Crude and age-specific incidence rates were computed using Poisson regression. The curves for parasite prevalence and incidence rate were fitted using locally weighted least squares (lowess) regression with standard errors estimated with 1000 bootstrapping replicates, with resampling at the subject level. Data analysis was performed in Stata, version 13 (Stata Corporation, College Station, TX) and R version 3.6.1 [R Core Team (2019), Vienna, Austria]. ArcMap version 10·5 (ESRI Inc., Redlands, CA, USA) was used to develop the map in Fig. 1.

## Results

### Community infection prevalence

Between March 2018 and February 2019, 6479 participants between the ages of 6 months–98 years were enrolled over four cross-sectional surveys. The overall RDT positivity, herein regarded as parasite prevalence (PR), across all four survey rounds was 9.9% (95% CI 9.2%, 10.7%) and was 13.7%, 10.7%, 7.2% and 9.0% during each cross-sectional survey, respectively (Additional file 1). PR did not differ during the wet (April, May, June, October, and November) *versus* dry seasons (9.5% vs. 10.4%; p = 0.264).

**Fig. 2:**
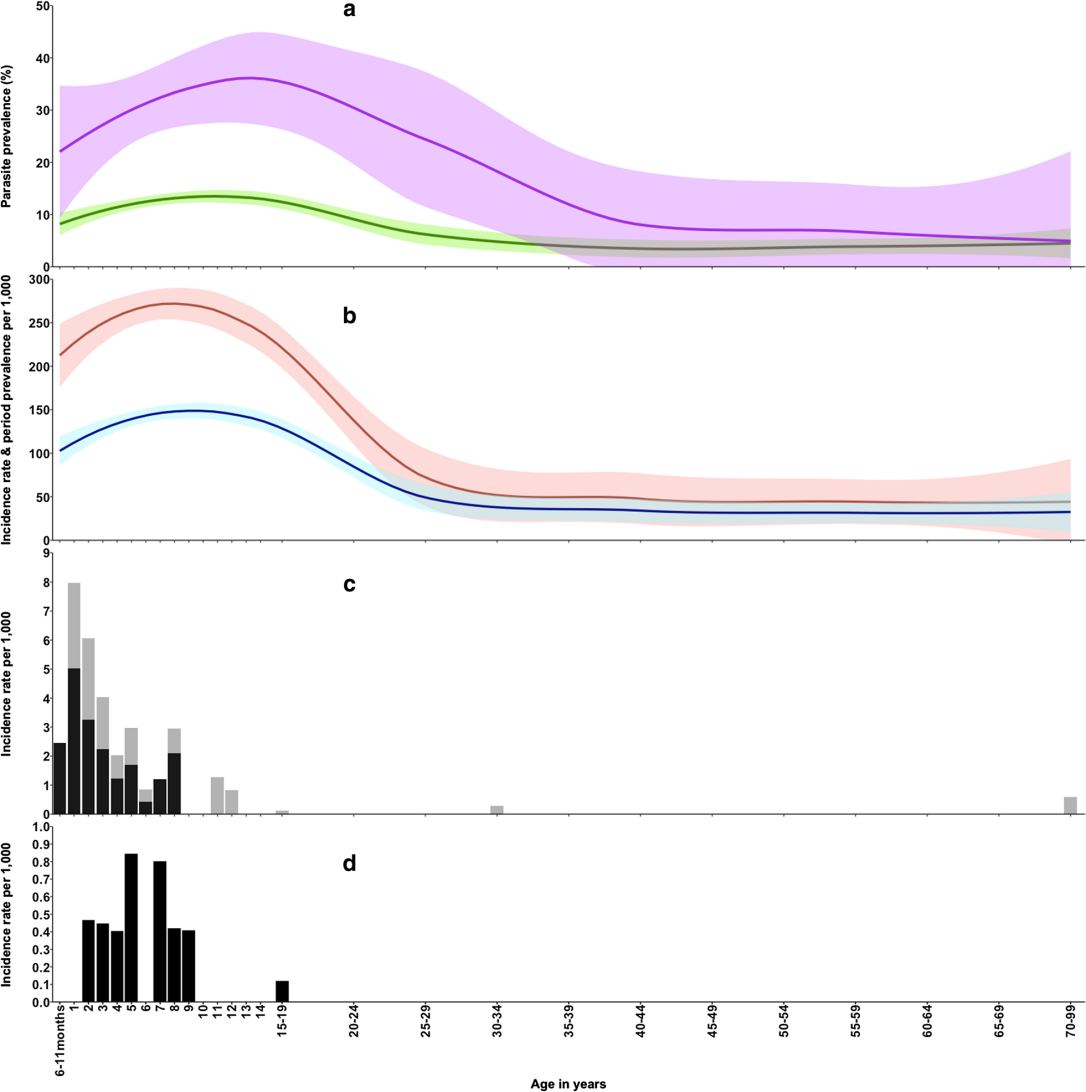
Comparison of malaria metrics across all age in the study area. **a** Parasite prevalence (green line) and community fever test positive prevalence (purple line). **b** The health facility RDT period prevalence (red line) and health facility RDT incidence rate (blue line). **c** The incidence of uncomplicated malaria admission (gray bar) and incidence of severe malaria (black bars). **d** Incidence of deaths attributable to malaria

Compared to children aged 6 months–4 years, children aged 5–14 years had a higher prevalence of malaria infection (PR = 13.4%; aOR = 1.28; 95% CI 1.01, 1.63; p < 0.001); after adjusting for site, month of enrollment, long lasting insecticide use (LLINs), and gender. Malaria parasite prevalence was lower in the older age groups compared to children aged 6 months–4 years (15–49 years—PR = 7.1%; aOR = 0.63; 95% CI 0.48, 0.82); with the lowest risk being reported among participants aged 50 years and above (PR = 4.3%; aOR = 0.38; 95% CI 0.23, 0.64). The age-pattern of PR showed a strong age-dependency, gradually increasing from childhood and reaching level of 12% by 9 years of age before declining from late adolescence into adulthood as shown in Fig. 2a and Table 1.

**Table 1:** Likelihood of malaria infection and disease severity by age group

Measure	6 months–4 years	5–14 years	15–49 years	50+ years	Overall
Parasite prevalence (PR)					
% (95% CI)	10.1% (8.5, 12.0)	13.4% (12.0, 14.9)	7.1% (6.0, 8.4)	4.3% (2.8, 6.4)	9.9% (9.2, 10.7)
n/N	136/1342	337/2523	146/2056	24/558	643/6479
Fever test positivity prevalence					
% (95% CI)	22.0% (13.8, 33.3)	35.0% (25.3, 47.1)	28.9% (18.9, 42.3)	11.4% (3.1, 29.3)	27.3% (22.7, 32.3)
n/N	22/200	43/123	26/90	4/35	95/348
Malaria passive period prevalence					
‰ (95% CI)	251.3‰ (241.8, 261.1)	271.0‰ (264.4, 277.7)	93.3‰ (89.8, 96.9)	47.8‰ (43.2, 52.8)	169.7‰ (166.9, 172.5)
n/N	2628/10,456	6388/23,571	2733/29,297	394/8238	12,143/71,562
Malaria passive incidence rate					
‰ (95% CI)	121.5‰ (114.9, 128.3)	150.1‰ (145.2, 155.2)	60.0‰ (57.3, 62.9)	33.4‰ (29.6, 37.6)	95.6‰ (93.5, 97.8)
n/N	1270/10,456	3539/23,571	1759/29,297	275/8238	6843/71,562
Malaria admissions					
‰ (95% CI)	4.7‰ (3.5, 6.2)	1.0‰ (0.7, 1.5)	0.07‰ (0.008, 0.2)	0.1‰ (0.003, 0.7)	1.1‰ (0.8, 1.3)
n/N	49/10,456	24/23,571	2/29,297	1/8283	76/71,562
Severe malaria					
‰ (95% CI)	2.9‰ (1.9, 4.1)	0.6‰ (0.3, 0.9)	0‰ (0, 0.1)	0‰ (0, 0.4)	0.6‰ (0.4, 0.8)
n/N	30/10,456	13/23,571	0/29,297	0/8283	43/71,562
Hospital malaria deaths					
‰ (95% CI)	0.1‰ (0.002, 0.5)	0.1‰ (0.03, 0.4)	0‰ (0, 0.1)	0‰ (0, 0.4)	0.06‰ (0.02, 0.1)
n/N	1/10,456	3/23,571	0/29,297	0/8283	4/71,562
Hospital + InterVA malaria Deaths					
‰ (95% CI)	0.4‰ (0.1, 1.0)	0.3‰ (0.1, 0.6)	0‰ (0, 0.1)	0‰ (0, 0.4)	0.1‰ (0.07, 0.3)
n/N	4/10,456	6/23,571	0/29,297	0/8283	10/71,562
Hospital + interVA + imputed malaria deaths					
‰ (95% CI)	0.6‰ (0.2, 1.2)	0.3‰ (0.09, 0.6)	0‰ (0, 0.1)	0‰ (0, 0.4)	0.2‰ (0.09, 0.3)
n/N	6/10,456	6/23,571	0/29,297	0/8283	12/71,562

*Parasite prevalence* was defined as the number of positive RDTs/total number of RDTs done in the community expressed per 100; *fever test positivity prevalence* was defined as the number positive RDTs/total number of community participants with fever (i.e. reported/measured fever); *malaria passive period prevalence* was defined as all cases testing RDT positive including re-attendance/person-years of observation expressed per 1000 individuals; *malaria passive incidence rate* was defined using records of first cases only of RDT positive patients/person-years of observation expressed per 1000 individuals; *malaria admission* was defined as number of episodes of malaria admissions/person-years of observation; *severe malaria* was defined as number of episodes of severe malaria admissions/person-years of observation (3 patients had been re-admitted to hospital with malaria each presenting twice and only one had severe malaria on both occasion); *hospital malaria deaths* was defined as the number of death during hospital admission attributable to malaria/person-years of observation; *hospital* + *interVA malaria deaths* was defined as the number of death attributable to malaria assigned either in the hospital or from the InterVA-4 model/person-years of observation; *hospital* + *InterVA malaria deaths* + *imputed malaria deaths* was defined as the number of death attributable to malaria assigned either in the hospital or from the InterVA-4 model or from multiple imputation/person-years of observation

Of the enrolled participants, 348 (5.4%: 95% CI 4.8%, 5.9%) had either measured or reported fever. Fever was associated with a higher risk of malaria infection (aOR = 5.2; 95% CI 3.9, 7.0; p < 0.001) compared to participants without fever; after adjusting for site, age, month of enrollment, LLINs use and gender.

### Passive case detection of fevers and infection

During the 12-month surveillance period, 28,134 febrile patients ≥ 6 months of age sought treatment in one of the six out-patient health facilities shown in Fig. 1. The largest number of attendees were among children aged 5–14 years (39%), with 26% of attendances among children aged 6 months–4 years, 28% among patients aged 15–49 years, and only 7% were aged 50 years and above.

The overall RDT positivity rate (TPR) was 43.2% (95% CI 42.6%, 43.7%). The median age of febrile patients who tested positive for malaria (10 years; IQR 5, 15 years) was significantly lower compared to the non-malaria fevers (12 years; IQR 4, 28 years) (p < 0.001). Febrile children aged 5–14 years had a higher risk of testing RDT positive (TPR = 57.9%; aOR = 2.40; 95% CI 2.21, 2.61; p < 0.001) compared to febrile children aged 6 months–4 years; after adjusting for site, month of enrollment, LLINs use, and gender. Other age groups had a lower risk of RDT positivity (i.e. for 15–49 years, TPR = 35.2%; aOR = 0.99; 95% CI 0.90, 1.08; and 50 years and above, TPR = 19.0%: aOR = 0.40; 95% CI 0.35, 0.46) compared to febrile children aged 6 months–4 years. TPR did not differ during the wet versus dry season (i.e. 42.7% vs. 43.5%; p = 0.173).

During the surveillance period there were 71,562 person-years of observation in residents of the study area aged ≥ 6 months. The annual period prevalence and incidence rate of RDT positivity followed a similar trend to that of infection prevalence in the community; initially increasing from childhood, plateaued in school-aged children (7–13 years) and peaked around 10 years before declining in late adolescence and adulthood (Fig. 2b). The incidence was lowest at 33.4/1000 per year among the elderly and highest at 150.1/1000 per year among children aged 5–14 year (Table 1).

### Hospitalized malaria and severe malaria

There were 597 in-patient admissions to Kilifi county hospital between March 2018 and February 2019 from the study area, 45% (n = 268) were female and 13% (n = 80) had been admitted in the paediatric high dependency unit (HDU). The overall median age of all-cause admission was 12 years (IQR: 2 years, 39 years). Among the 76 malaria admissions, the majority (57%) presented with one or more criteria for severe malaria; 33 (43%) cases had uncomplicated malaria. The ratio of children with cerebral malaria to severe malaria anaemia was 5:2. There were no cases of severe malaria among patients aged 9 years and above.

The age-pattern of incidence of malaria admissions is shown in Fig. 2c and Table 1. Incidence of malaria admission was highest among children aged 1 year old (7.96 per 1000 person-years: 95% CI 4.79, 12.43 per 1000 person-years), and then declined thereafter. Malaria admission among adults was negligible. A similar trend was observed with the severe malaria cases, where severe malaria cases peaked among children aged 1 year (5.03 episodes per 1000 person-years: 95% CI 2.60, 8.78 episodes per 1000 person-years) and then sharply declined thereafter (Fig. 2c).

### Malaria mortality

A total of 316 deaths were recorded among the population of the study area between March 2018 and February 2019. Thirty-six deaths (11%) were among children aged 6 months–4 years, 22 (7%) among 5–14 years, 61 (20%) among 15–49 years and 197 (62%) deaths among those aged 50 years and above.

Of the 316 deaths, 55 (17%) died during hospital admission including 3 malaria deaths and 8 (2.5%) died < 30 days post-discharge with one diagnosed with malaria during admission. Four (1.3%) individuals had been admitted over the preceding 24 months, where three had been diagnosed with terminal cancer and one with epilepsy (Additional file 2). VA interviews were completed for 197 (79%) of 249 deaths without hospital contact. The median time to VA interview was 10.2 weeks (IQR: 6.9 weeks, 14 weeks). 35 deaths were classified as unambiguous causes of death that were not malaria after reviewing the InterVA-4 100% probability assigned cause or available information in the free text fields (Additional file 2). VA data was missing for 52 (16%) of 316 deaths. There was no difference in the age distribution of complete cases *versus* those missing VA data (p = 0.09). Using the MICE algorithm, there were 2 cases (IQR 1, 3 cases) aged 6 months–4 years ascribed malaria based on the distribution of deaths among known causes.

Malaria was attributed as the primary cause of death among 6/36 children aged 6 months–4 years, 6/22 deaths among children aged 5–14 years, and none among the 258 adult deaths above 15 years (Table 1; Fig. 2d). Only 3.7% (12/316) of deaths were attributable to malaria. Malaria mortality rates among children aged 6 months–4 years and 5–14 years, were 0.57 per 1000 person years (95% CI 0.2, 1.2) and 0.25 per 1000 person-years (95% CI 0.09, 0.56), respectively (Table 1).

## Discussion

Paramount to the understanding of the expected impact of interventions that reduce parasite exposure is the relationship between malaria transmission intensity and the ensuing burden of disease, or mortality, and how this varies with age. Empirical data on the incidence of new infections, mild clinical disease, severe disease and death in a single location remain rare, and generally focus on childhood. Little is currently known about these relationships in non-pregnant adults.

### Malaria infection prevalence

Across four cross-sectional surveys the overall prevalence of infection was 10%, without significant seasonal variation. Using a previously developed mathematical model on the relationship between prevalence in children aged 2–10 years and entomological inoculation rates (EIR) [46], it was estimated that individuals in this area might expect to receive one infectious bite every 2 years.

**Fig. 3:**
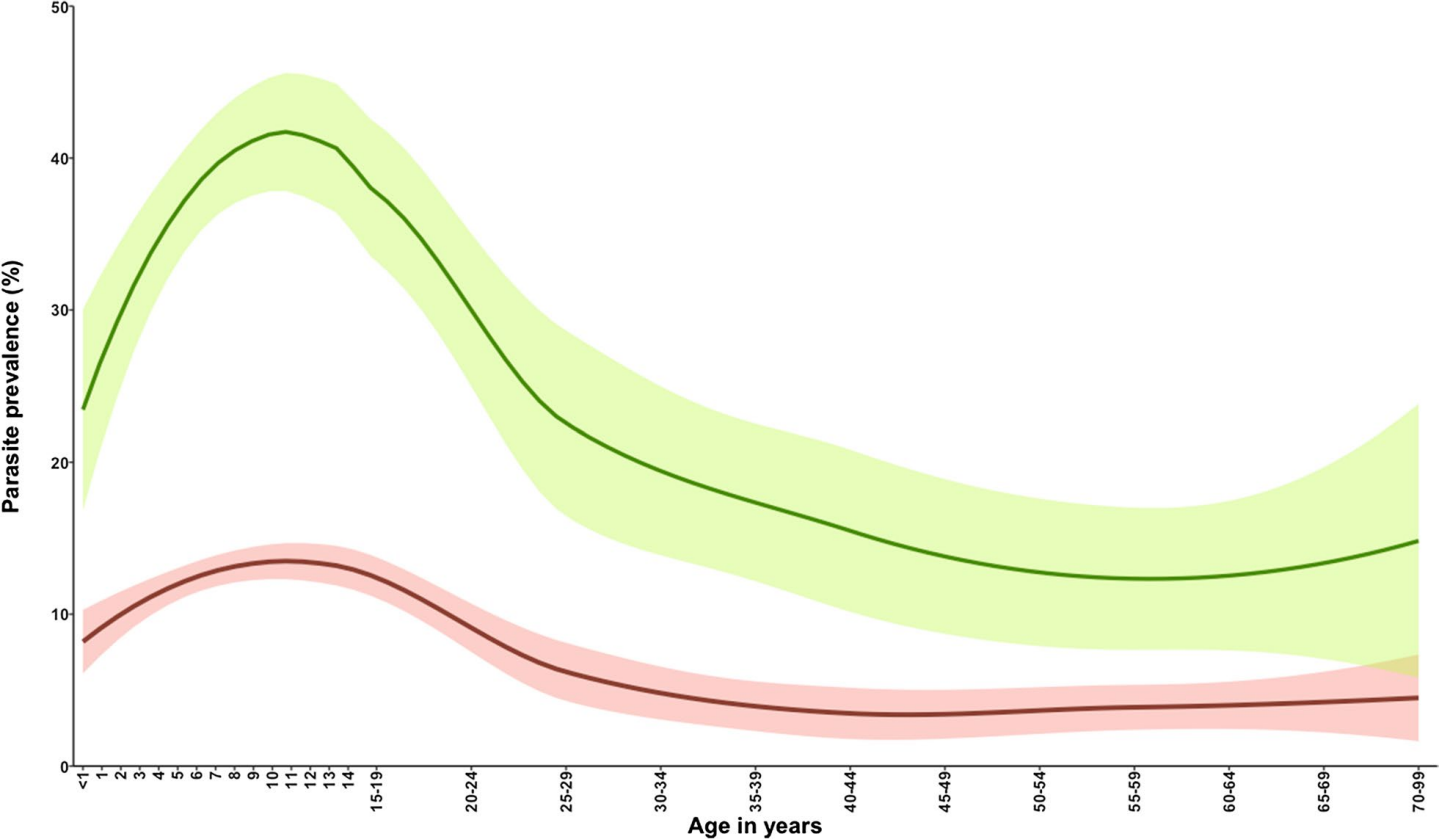
Parasite prevalence between 1999 and 2001 (green line) [24, 48] and current parasite prevalence between 2018 and 2019 (red line)

Despite a low transmission intensity, the age-pattern of infection was consistent with patterns previously described across a wide range of endemicities [28, 47]. Infection rose through childhood to peak at 12 years, before declining after 24 years and remained relatively constant, below 5%, throughout adult life (Fig. 2a). Previous studies in the same area of the Kenyan coast undertaken 20 years ago [24, 48] showed a substantially higher parasite rate of 43.1%. However, the age-pattern of infection was similar, the peak infection prevalence was at 10 years with a slightly more rapid decline after 19 years of age, suggesting that although infection was less frequent, the acquisition of resistance to infection is acquired at a quite similar rate (Fig. 3).

From the age of 30 years, prevalence steadily decreased to very low levels through to old age, suggesting a continued acquisition of immunity to infection throughout adulthood (Fig. 2a), similar to what was observed 20 years ago in the same area (Fig. 3). This pattern was also observed among adults tested with PCR in Mozambique [49]. It is unclear whether the immunity to infection is sterilizing, or simply suppresses parasite densities below the threshold of detection.

### Incidence of fevers associated with malaria

Over 12 months, 12,143 fevers associated with malaria infection presented to the six health facilities shown in Fig. 1, among a census population of 71,562 aged 6 months and above. This corresponded to a 95.6 (95% CI 93.5, 97.8 episodes per 1000 person-years) annual incidence of passively detected cases of malaria per 1000 person-years per annum.

The age-pattern of the period prevalence and incidence of fevers associated with malaria rose in childhood to peak at 10 years and declined thereafter to a low level throughout adulthood (Fig. 2b). The highest risk in children aged 5–14 years was consistent with the background prevalence of infection in this community and may represent coincidental infection rather than a causally linked febrile episode in this age group. However, among all individuals presenting to the health facilities with RDT positive fevers, 52.6% were aged 5–14 years and therefore represents a substantial health system burden requiring malaria treatment according to national guidelines [50–55].

### Severe malaria

The age-specific incidence of severe malaria showed a very different age-profile to either community-based or passively detected fever prevalence of malaria infection. The incidence of hospitalized malaria from the same community was concentrated among children aged 6 months to 4 years. There were three cases of non-severe, hospitalized malaria described in adults aged, 18, 31, 88 years, none of these presented with severe malaria (Fig. 2c). Despite the low numbers of severe malaria cases from the specified study area, cerebral malaria was most prevalent syndrome compared to severe malaria anaemia. At current levels of low infection prevalence, and presumed EIR, hospitalized malaria continues to be a paediatric problem, with the median age of admission being 37 months among children aged less than 15 years.

The present study area has also been subject to a transition in the intensity of malaria transmission over the last 20 years [24, 48]. This is important when considering risks among adults, who will have been exposed to very different levels of parasite exposure during their childhood. Nevertheless, the declining incidence over the first 5 years of life (Fig. 2c), when transmission intensity is likely to have remained constant, still suggests that immunity to severe disease is still acquired early in life. The rarity of malaria requiring hospitalization and the absence of severe malaria after 8 years suggests that few infections, early in life might confer a long-term protection against severe, life-threatening morbidity [56]. The data suggest that even when malaria transmission falls significantly, immunity is still acquired.

### Malaria mortality

During the study period there were 316 deaths recorded within the community aged 6 months and above. The overall malaria-specific mortality rate was estimated to be 0.17 per 1000 population above 6 months p.a. (95% CI 0.09, 0.29 episode per 1000 person-years). With this approach, there were no malaria deaths among children 6–11 months of age, 6 (50%) of the malaria deaths occurred among children aged 1–4 years, 6 (50%) among children aged 5–14 years, and there with no deaths attributed to malaria among the non-pregnant population aged above 15 years (Table 1; Fig. 2d). Fatal malaria events are still concentrated in young children.

The Global Burden of Disease (GBD) project relies on VA data from DSS sites in sub-Saharan Africa, which have shown different malaria-specific age patterns [57–59]. Between 1980 and 2010 the percentage of deaths attributable to malaria among individuals aged ≥ 15 years was 58% in sub-Saharan Africa, more than those defined among children < 5 years of age (24%) [59].

In the absence of clinical and parasitological data, the definition of mortality events directly attributed to malaria are fraught with difficulties [60], leading to misclassification and inconsistent results [61–63]. Malaria is principally a febrile illness, associated with many co-presenting symptoms, including respiratory complications, vomiting, altered consciousness and seizures. These symptoms are often associated with other common causes of death within the same community and age groups. The recent introduction of minimally invasive autopsy techniques [64], might offer further opportunities to explore the true contribution of malaria to deaths in non-pregnant adults in Africa.

Deaths that had contact with diagnostic facilities provide unique information on causes of death. Relying solely upon a computerized algorithm to attribute malaria as a cause of death from VA responses misses opportunities to review underlying causes available in free-text commentaries, notably in the identification of important unambiguous causes, including HIV and SCD. The approach taken here maximizes the amount of information available, and by comparing with hospital presentations with severe malaria, allows a more plausible approach to defining the contribution of malaria mortality by age.

### Limitations

Here RDTs have been used to describe prevalence of HRP2 antigenaemia, to serve as a direct comparison with methods used during surveillance at health facilities. However, RDTs are often described as having a high false positive rate compared with microscopy as the HRP2 antigenaemia detected by RDTs may reflect recent infection that has been cleared. An analysis of these imperfect tests using a Bayesian framework applied to data from Kilifi [65] concluded that microscopy and RDT afford high sensitivity and specificity in symptomatic care-seeking children in this low *P. falciparum* prevalence setting. Nevertheless, RDTs are now widely used in clinical and survey settings, but it is important to note that comparisons with other data on microscopy (Fig. 3) are relative, rather than absolute. Secondly, this study did not include children under 6 months of age because including this age group would be considered as a different study question regarding passive immunity and it would require staff to be trained further on how to collect a blood sample. However, it seems unlikely that measures reported among children under-5 years would be significantly different from those reported among children aged 6 months–4 years. Thirdly, during the hospital surveillance, not all adults had a blood slide for malaria performed which could have led to an under-estimation of incidence of hospitalized malaria among the adult population. However, it is unlikely that the general conclusion would have changed significantly, since 89% (250/280) of all adult admissions had a chronic illnesses or final diagnoses that included skin/organ infections, trauma, hernia or obstetric related problems. Of the remaining 30 infectious disease admissions, 23 (77%) had a blood film to confirm malaria. Fourthly, although the age pattern of severe disease and mortality are shown in this study, caution should be used in interpreting the patterns observed because these are rare events which would require a larger population to interrogate the finer age-resolved patterns for both outcomes. Additionally, the number of deaths and severe disease reported here may not be representative of all cases of malaria, as an unknown proportion of cases never present to the formal health care system. Lastly, this study was conducted in a single study site and the results may not be generalizable to other settings.

## Conclusions

Despite a reduction in the transmission intensity of malaria over the last 20 years, immunity to severe and fatal consequences of *P. falciparum* continue to be acquired in childhood, faster than anti-parasitic immunity. There is no evidence of an emerging significant burden of severe malaria or malaria mortality among young adults. This is contrary to current modelled approaches to disease burden estimation in Africa. The continued absence of precise estimates of the age-specific malaria mortality patterns in the community limits the ability to look at immune protection against fatal outcomes of infection and presents a problem in defining the global and national burdens of malaria mortality [60, 66]. The results presented here support continued, targeted infection prevention strategies to prevent life threatening disease among young children in low transmission settings, while reductions in infection prevalence and mild disease would benefit from interventions that reach school-aged children.

## Supplementary information

**Additional file 1.** Seasonality of parasite prevalence using mRDTs (red bars) between May 2018 and January 2019 and total monthly rainfall over the 12-month surveillance period (black dashed line).

**Additional file 2.** Varying confirmatory evidence to attribute deaths.

## Data Availability

Data that support the findings of this study (verbal autopsy data, hospital surveillance, health facility and community surveys) are available from the KEMRI Institutional Data Access/Ethics Committee. Details of the guideline can be found in the KEMRI-Wellcome data sharing guidelines. The data includes homestead level coordinates as an essential component and these are personally identifiable data. Access to data is provided via the KEMRI Wellcome Data Governance Committee: dgc@kemri-wellcome.org; Tel, +254,708 587,210; Contact person, Marianne Munene (Secretary; Tel, +254,709 983,436).

## Abbreviations

OPD: Outpatient department
HRP2: Histidine rich protein 2
RDT: Malaria rapid diagnostic test
SSA: Sub Saharan Africa
DSS: Demographic surveillance system
EZ: Enumeration zone
KCH: Kilifi County Hospital
COD: Causes of death
VA: Verbal autopsy
SCD: Sickle cell disease
WHO: World Health Organization
MICE: Multivariate imputation by chained equations
aOR: Adjusted odds ratios
PR: Parasite prevalence
TPR: Test positivity rate
IQR: Interquartile range
EIR: Entomological inoculation rates
HIV: Immunodeficiency virus
GBD: Global Burden of Disease
LLIN: Long lasting insecticide net
