# Supplementary material for: Malaria infection, disease and mortality among children and adults on the coast of Kenya
Source: Malar J. 2020 Jun 17;19:210. doi: 10.1186/s12936-020-03286-6 (PMC7301992; doi:10.1186/s12936-020-03286-6)
Supplement: Supplementary file 1 — Additional file 1. Seasonality of parasite prevalence using mRDTs (red bars) between May 2018 and January 2019 and total monthly rainfall over the 12-month surveillance period (black dashed line). [file 12936_2020_3286_MOESM1_ESM.docx]

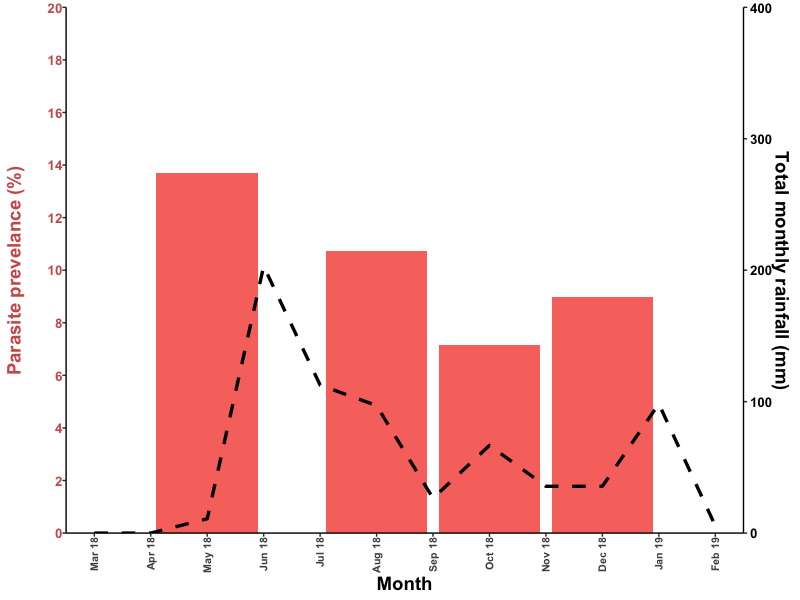


**Additional file 1: Seasonality of parasite prevalence using mRDTs (red bars) between May 2018 and January 2019 and total monthly rainfall over the 12-month surveillance period (black dashed line).**

Between May and June 2018, 1,445 participants were approached for enrolment; 32 (2.2%) declined consent and 69 were excluded because they either had been enrolled in the health facility survey within the last 14 days, were pregnant or had missing mRDT results. 1,922 participants were approached for enrollment in August 2018; 168 (8.7%) declined consent and 46 were excluded for reasons stated above. In October 2018, 1,895 participants were approached for enrollment; 99 (5.2%) declined consent and 62 were excluded as stated above. Between December 2018 and January 2019, 1,852 participants were approached for enrollment; 79 (4.3%) declined consent and 80 were also excluded.
