# Supplementary material for: Malaria infection, disease and mortality among children and adults on the coast of Kenya
Source: Malar J. 2020 Jun 17;19:210. doi: 10.1186/s12936-020-03286-6 (PMC7301992; doi:10.1186/s12936-020-03286-6)
Supplement: Supplementary file 2 — Additional file 2. Varying confirmatory evidence to attribute deaths. [file 12936_2020_3286_MOESM2_ESM.docx]

**Additional file 2: Varying confirmatory evidence to attribute deaths.**
